# An Image Encryption Scheme Based on Block Scrambling, Modified Zigzag Transformation and Key Generation Using Enhanced Logistic—Tent Map

**DOI:** 10.3390/e21070656

**Published:** 2019-07-03

**Authors:** Priya Ramasamy, Vidhyapriya Ranganathan, Seifedine Kadry, Robertas Damaševičius, Tomas Blažauskas

**Affiliations:** 1 Department of Applied Mathematics and Computational Sciences, PSG College of Technology, Coimbatore, TamilNadu 641004, India; 2 Department of Biomedical Engineering, PSG College of Technology, Coimbatore, TamilNadu 641004, India; 3 Department of Mathematics and Computer Science, Beirut Arab University, Beirut 11-5020, Lebanon; 4 Department of Software Engineering, Kaunas University of Technology, Kaunas 51386, Lithuania

**Keywords:** image encryption, chaotic system, block scrambling, modified zigzag transformation, 3D logistic map, entropy analysis

## Abstract

Nowadays, the images are transferred through open channels that are subject to potential attacks, so the exchange of image data requires additional security in many fields, such as medical, military, banking, etc. The security factors are essential in preventing the system from brute force and differential attacks. We propose an Enhanced Logistic Map (ELM) while using chaotic maps and simple encryption techniques, such as block scrambling, modified zigzag transformation for encryption phases, including permutation, diffusion, and key stream generation to withstand the attacks. The results of encryption are evaluated while using the histogram, correlation analysis, Number of Pixel Change Rate (NPCR), Unified Average Change Intensity (UACI), Peak-Signal-to-Noise Ratio (PSNR), and entropy. Our results demonstrate the security, reliability, efficiency, and flexibility of the proposed method.

## 1. Introduction

With rapid spread of cloud computing, mobile networks, internet of things, and social networking, the issue of secure transmission of image data has become increasingly relevant [1,2]. Encrypting secret information that is sent over Internet or wireless networks as multimedia is important in satisfying the need of a secure route of data transmission over various communications channels. Averting unapproved access, adjustment, or the destruction of data ought to anchor the data exchanged through these channels. Different types of data are transmitted over the channels, for example, text, images, audio, video, three-dimensional (3D), and others for many domains of application, such as military, medical, financial institutions, etc. However, the security of those images is a challenge. The critical part of exchanging images is security, in order to protect the image from unauthorized access and modification.

The utilization of cryptographic strategies for image encryption is especially required in order to provide a powerful solution to the security of images. The cryptographic strategies convert the image into an irrelevant data sequence that cannot be effortlessly broken by the attacker. The target of image encryption is to provide high security and avoid unauthorized access. Standard cryptography techniques, such as Advanced Encryption Standard (AES), are regularly applied for text messages. However, those techniques, due to specific qualities, for example, extensive information and high correlation among image pixels, are not reasonable for media data.

The essential methods for an encryption framework can be arranged into two fundamental classes: diffusion and confusion [3]. The chaotic sequence creates the mapping with random sequence. These structures are excessively unpredictable and hard to break down and anticipate [4,5]. Previously, various encryption techniques that are dependent on chaos have been examined and broadly contemplated. Image encryption algorithms have been constructed based on a logistic and two-dimensional (2D) chaotic economic map [6], variable length codes that are based on Collatz conjecture [7], 2D discrete wavelet transform and Arnold mapping [8], logistic mapped convolution and cellular automata [9], cat map [10], 2D Chebyshev-sine map [11], 2D Sine Logistic modulation map [12], one-dimensional (1D) delay with linearly coupled Logistic chaotic map [13], a hyper-chaotic system that combines Dynamic Filtering, DNA computing, and Latin Cubes (DFDLC) [14], Arnold Transform followed by Qubit Random Rotation [15], 2D Baker’s map with diffusion process based on XORing [16], ant colony optimization [17], Chebyshev Map followed by Rotation Equation [18], an algorithm combining Julia fractal and Hilbert curve [19], four-dimensional (4D) hyper-chaotic nonlinear Rabinovich system [20], Josephus traversing and mixed chaotic map [21], 2D logistic-modulated-sine-coupling-logistic chaotic map [22], multiple permutation of pixels followed by the 2D Chebyshev function [23], chaos map with pixel permutation [24], improved hyperchaotic sequences [25], high-dimension Lorenz chaotic system with a perceptron model [26], rotation matrix bit-level permutation with block diffusion [27], and discrete Chirikov map with chaos-based fractional random transform [28].

Cryptanalysis is the science of deciphering secret keys or plaintext when compared with cryptography [29,30,31,32,33], comprising a further branch of cryptology. Research on cryptanalysis is of high importance in promoting cryptology advancement. Applying insecure algorithms for communication will result in severe security threats and losses on both ends of communications if security bugs are not found in encryption cryptosystems. The latest studies have demonstrated that some image encryption methods that are based on chaos schemes have vulnerabilities. Li et al. [29] used the chaotic tent map (CTM) with the diffusing phase only, while the confusing phase was skipped. Consequently, the CTM based image encryption has security defects. Wu et al. [30] used CTM with rectangular transform. The scheme included confusion and diffusion, followed by an improved 2D Arnold transform, which thus improves the security of the classical CTM based method. Wu’s algorithm has the advantages of easy design, high encryption speed, and good cryptographic efficiency as a typical colour image encryption method is concerned. However, it cannot resist the chosen plaintext attack, and the encryption method is insensitive to all secret keys. Li et al. [31] evolved the cipher text-only, known-plaintext, and chosen-plaintext attacks on the Ye’s scheme [24]. Li et al. [32] created the known-plaintext attack on the Zhu’s system [25]. Guo et al. [33] used the equivalent key attack on the 3D chaotic Baker map based image cryptosystem. Some of the chaos-based image encryption schemes that have been broken are discussed in [34]. Cryptanalysis can also assist developers to enhance the safety of the encryption algorithm, in addition to revealing weaknesses in encryption algorithms. Sam et al. [35] used shift rotate within the chaotic framework, which allowed for adaptability proficiency, straightforwardness, and resistance against known assaults. Wang et al. [36] added a shift operation to Huang’s scheme [23], which prevents the recovery of the shuffle vectors, thus increasing the security against the chosen-plaintext attack, but without a noticeable loss of efficiency. Wang et al. [37] offered an encryption technique that used logistic mapping and the combined row and scrambling technique to improve the security characteristics. 

So far, few results depending on confusion and diffusion have been suggested [38,39,40,41,42,43], providing an understanding that there is a solid connection between chaos and cryptography. The chaotic behavior system framework ensures high efficiency and high safety due to pseudo-randomness, as they are sensitive to initial conditions. The one-dimensional (1D) chaotic systems, such as pseudo-randomly enhanced logistic map (PELM) [44], are more attractive than multi-dimensional (MD) chaotic systems in order to create pseudo-random keystream [45], due to high speed and simplicity. However, it still has some limitations, such as discontinuity, numerical degradation, non-consistency, and weak key space [46], which thus motivates the need for MD maps, such as a3D mixed chaotic map [47]. Articles [48,49] suggested the intertwining of the logistic maps to strengthen the security and to enhance pseudo-randomness and increase the key space. 

Here, we propose a novel key generation algorithm that uses block scrambling, modified zigzag transformation, and enhanced logistic–tent map for image encryption. The current work extends we work of Li et al. [50], by suggesting the use of Enhanced Logistic–Tent Map (ELTM) instead of 3D logistic map to obtain better encryption characteristics. The principal contribution of the paper is the proposed key generation algorithm using ELM, which provides high security, as it takes plain image and key for each iteration of key generation. Here, a new efficient ELTM bases algorithm is proposed. The statistical test analysis is done by the National Institute of Standards and Technology (NIST) statistical test suite demonstrates its better security characteristics.

The remaining parts of the paper are as follows. We describe the methods used in Section 2. In Section 3, the results are presented. In Section 4, the NIST statistical test suite results are given. Finally, we summarize our results and present the conclusions in Section 5.

## 2. Materials and Methods

### 2.1. Logistic Map

Chaotic maps are highly sensitive to the initial value, which makes them unpredictable. A change in the numerical sequence that is generated by the function can occur, even if a minor change in the initial value is executed [51]. Different forms of chaotic maps are used; however, the logistic map is perhaps the most known map and is defined in Equation (1) [13], as follows:

(1) $$T_{n+1}=rT_{n}\left(1-T_{n}\right),$$

here *T_n_* is the state, *r* is the behavior parameter, and *n* is the count of iterations used to generate the state values iteratively.

This 1D logistic map can be extended to a three-dimensional (3D) one, as indicated in Equation (2) [52].

(2) $$\begin{array}{l} T_{n+1}=\alpha T_{n}\left(1-T_{n}\right)+\lambda U_{n}^{2}T_{n}+\beta V_{n}^{3}\\ U_{n+1}=\alpha U_{n}\left(1-U_{n}\right)+\lambda V_{n}^{2}U_{n}+\beta T_{n}^{3}\\ V_{n+1}=\alpha V_{n}\left(1-V_{n}\right)+\lambda T_{n}^{2}V_{n}+\beta U_{n}^{3} \end{array}$$

If the values of the parameters fall within the ranges 0.53 <α < 3.8, 0< λ < 0.022, 0 < β < 0.015, here $T_{0},\;U_{0},\;V_{0}$ are in [0, 1], and then the chaotic behavior is observed.

### 2.2. Skew Tent Map

The skew tent map is represented by the nonlinear equation [52]:

(3) $$T_{n+1}=\{\begin{array}{c} T_{n}/c,0<T_{n}\leq c\\ \left(1-T_{n}\right)/\left(1-c\right),c<T_{n}<1 \end{array},$$

here $T_{n}\in \left[0,1\right]$ is the state, c∈[0,0.5]∪[0.5,1] is the behaviour parameter, and *n* is the count of permutations that are used to create state values iteratively.

### 2.3. Block Scrambling

First, the RGB image, which has the dimension of 256×256×3 is segregated into four quadrants. Subsequently, each quadrant is further segregated into four sub-quadrants, while each sub-quadrant is rotated anti-clockwise by 90° to form 64 sub-blocks. While this procedure scrambles the image, it does not fully remove the associations between the nearby pixels. 

### 2.4. Modified Zigzag Transformation

Zigzag Transformation (ZT) is a procedure to have an image scrambled [53]. The red, green, and blue image channels are treated as separate matrices having the size of 256×256 pixels. In ZT, the upper left pixel is shifted to one side, which allows for the attacker to crack the strategy. In the modified ZT, the upper left and next horizontal neighboring pixels are exchanged with the base right pixel. 

The change is performed to the pixels of every matrix, starting from the upper left corner and ending with the base right corner to execute encryption. The first and the second elements of the matrix are moved to the last and one-before-last position of the matrix, and the remaining elements are swapped in a zigzag way. 

The first and second elements of the matrix are moved to the last and the one-before-last elements of the matrix and the remaining elements of the matrix are swapped in a zigzag way to execute decryption. The change is connected to the pixels of every framework as delineated. This strategy totally twists the relationship among the nearby pixels of the image, which results in the modified ZT of the image.

### 2.5. Enhanced Logistic Map (ELM)

The proposed Enhanced Logistic Map (ELM) enhances the security of the scheme by employing the chaotic diffusion method. The ELM is 3D, as it separately deals with RGB of the color image. The ELM is defined, as follows:

(4) $$\begin{array}{l} X_{i+1}=-\lambda T_{i}\left(1-X_{i}\right)+\beta Y_{i}^{2}X_{i}+\alpha Z_{i}^{3}+c\\ Y_{i+1}=-\lambda U_{i}\left(1-Y_{i}\right)+\beta V_{i}^{2}Y_{i}+\alpha X_{i}^{3}+c\\ Z_{i+1}=-\lambda Z_{i}\left(1-Z_{i}\right)+\beta X_{i}^{2}Z_{i}+\alpha Y_{i}^{3}+c \end{array}$$

where, λ, β, α, c are the constants, 0.8<λ<2.60, 0<β<0.15, 0.42<α< 0.85, 0<c<0.35. The range of all λ, β, α, c are greater than the 3D logistic map, thus it provides better security than the 3D logistic map. 

**Figure 1:**
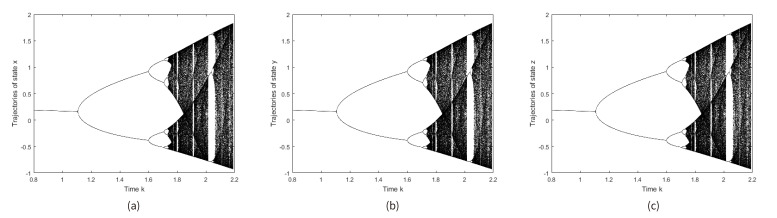
Bifurcation diagram of x, y, and z values for Enhanced Logistic Map (ELM): (**a**) x values, (**b**) y values, and (**c**) z values.

**Figure 2:**
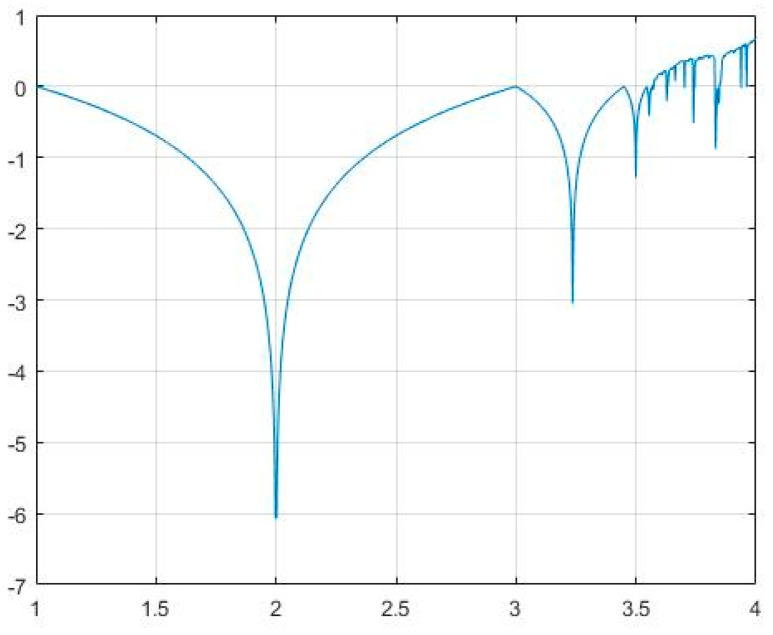
Lyapunov Exponent of Enhanced Logistic Map.

The bifurcation diagram and the Lyapunov Exponent of ELM are shown in Figure 1 and Figure 2, respectively.

### 2.6. Key Generation

Both encryption and decryption processes use the same key. $X_{0},\;Y_{0},\;Z_{0}$ have their values in [0, 1]. Accordingly, the values are transformed to the range 0–255. The equation for generating the values of X, Y, Z are presented below:

(5) $$\begin{array}{l} X_{i}=\left\lfloor 10^{14}\left(X_{i}\right)\mathrm{mod}256\right\rfloor \\ Y_{i}=\left\lfloor 10^{14}\left(Y_{i}\right)\mathrm{mod}256\right\rfloor \\ Z_{i}=\left\lfloor 10^{14}\left(Z_{i}\right)\mathrm{mod}256\right\rfloor \end{array}$$

For example, the initial values for $X_{i},Y_{i},Z_{i}$ are defined as $X_{0}=\;0.790$, $Y_{0}=\;0.889$, and $Z_{0}=\;0.590$, respectively, and the values of constants are λ=2.741, β=0.021,α=0.041, and c= 0.9.

The cryptosystem is secured with the plain image, the novel proposed system employs a 256-bit key (K), which has 192-bit data calculated from ELM, the secret key ($K_{e}$), and 64-bit data chosen by the user from a plain image ($K_{d}$). $K_{d}$ retains pixels $R\;\left(r_{i},\;r_{j}\right),\;G\;\left(g_{i},\;g_{j}\right),\;B\;\left(b_{i},\;b_{j}\right)$, which are selected by the encoder. Now, $K\;=\;K_{e}K_{d}$, where the key *K* is segregated into 16-bit parts $ks_{1},\;ks_{2}\ldots \;ks_{16}$. 

The initial condition $I_{c}$, the parameter $P_{x}$ and the iteration count n of the skew tent system are defined by the following equations:

(6) $$\begin{array}{l} \begin{array}{l} I_{c}=\;ks_{2}⊕ks_{4}⊕\cdots ⊕ks_{14}⊕ks_{16})/256\;\\ P_{x}=ks_{1}⊕ks_{3}⊕\cdots ⊕ks_{9}⊕ks_{11})\;+ks_{13}+ks_{15})/758 \end{array}\\ n=(ks_{14}⊕ks_{15})+ks_{16}\; \end{array}$$

As a result, the generated key depends on the image and EX-OR operation is executed with each value of R, G, B.

### 2.7. Encryption Algorithm

The proposed framework can be used for any M × M color image. Encryption has two parts: confusion and diffusion. Blocks scrambling is used to obtain 64 squares when confusion is performed. Finally, the modified ZT is performed to remove the association between the adjacent pixels. Key generation executed while using the 3D ELM to produce secret keys in the range 0–255 for every X, Y, and Z. In the final step, the EX-OR operation is executed to obtain the cipher image. 

Algorithm 1 summarizes the algorithm of encryption.

**Algorithm 1:** Algorithm of the encryption processInput: plain color image P of size 256×256
Output: cipher image C
**Step 1:** Block scrambling is applied on P, which is split into 64 blocks each of size 32 × 32 represented as $C_{1}$. **Step 2:** Modified zigzag transform (ZT) is performed on the scrambled blocks $C_{1}$ to obtain $C_{2}$. **Step 3:**
$C_{2}$ is split into RGB channels each of size 256×256. **Step 4:** Using ELM, the intermediate key is generated with initial values are taken as $X_{0}=0.790,\;Y_{0}=0.889,\;Z_{0}=0.590$, respectively. **Step 5:** The final key is generated by applying the chosen values from image and external user as initial condition and parameters. **Step 6:** The secret key K is EX-OR-ed with the RGB channels received after modified ZT to obtain C.

**Figure 3:**
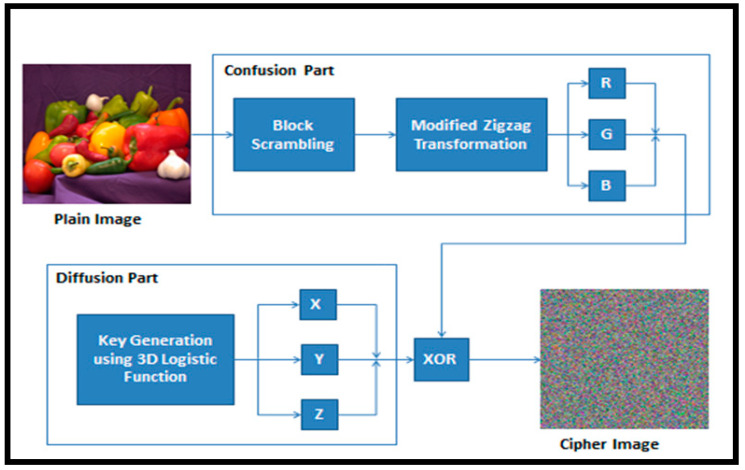
Block diagram of encryption process.

Figure 3 presents the block diagram for the encryption procedure. The decryption process is performed in reverse of encryption to obtain plain image P. 

### 2.8. Evaluation

We assess the security characteristics of the proposed scheme while using histogram analysis, information entropy analysis, correlation coefficient, Number of Pixels Change Rate (NPCR), Unified Average Change Intensity (UACI), Mean Square Error (MSE), and Peak Signal to Noise Ratio (PSNR).

#### 2.8.1. Key Space Analysis

The proposed method is heavily dependent on the key, it should ensure that the key is secure, and that the key space should be adequately large to make the brute force attack impossible. As the proposed algorithm uses a 256-bit key, the number of admissible secret key combinations is 2^256^ for R, G, and B separately, making it quite difficult to break while using brute force.

#### 2.8.2. Key Sensitivity Analysis 

The good encryption method must be very sensitive to changes in the keys. Implementing a small change to the encryption key, the output image must be very different when compared to the unmodified encrypted image.

#### 2.8.3. Histogram Analysis

The histogram represents the distribution of pixel values in an image. An encrypted image is expected to have a uniform distribution of the histogram values, making for the attacker difficult to learn something about the image. Thus, the suitability of the proposed encryption method is shown by the uniform distribution of pixel values in a coded image. 

#### 2.8.4. Correlation Coefficient Analysis

Usually, the neighboring pixels of the plain image are related, while the adjacent pixels of the encrypted image are weakly correlated, which suggests that there are no associations between them. 

Correlation Coefficient Analysis (CCA) is performed to assess the level of similarity between the pair of pixels. This involves the calculation and assessment of the Pearson correlation coefficient alongside the vertical, horizontal, and diagonal directions of both the plain and encrypted image. 

A good encryption method should break the correlation between adjacent pixels. The less the correlation is, the more effectively the method performs. The correlation coefficient is calculated, as follows:

(7) $$\begin{array}{l} CR=\frac{\mathrm{cov}\left(X,Y\right)}{\sqrt{D\left(X\right)}\sqrt{D\left(Y\right)}}\\ \mathrm{cov}\left(X,Y\right)=\frac{1}{256}\sum_{1}^{256}\left(X_{i}-E\left(X\right)\right)\left(Y_{i}-E\left(Y\right)\right) \end{array},$$

here *X* and *Y* are the pixels and neighboring pixels of the original and encrypted image, cov(X,Y) is the covariance between X and Y, D(X) is variance of X, and E(X) is the expected value of X.

#### 2.8.5. NPCR and UACI Analysis

Number of Pixels Change Rate (NPCR) assesses the pixel difference between the original and encrypted images [54,55], as follows:

(8) $$NPCR=\frac{\sum_{i,j}D\left(i,j\right)}{MN}\cdot 100,$$

here D(i,j) is calculated as

(9) $$D\left(i,j\right)=\{\begin{array}{cc} 0 & P\left(i,j\right)=C\left(i,j\right)\\ 1 & P\left(i,j\right)\neq C\left(i,j\right) \end{array},$$

Higher randomization of the pixel values leads to a larger value of NPCR. 

The Unified Average Changing Index (UACI) assesses the mean intensity of differences between the original image and encrypted image [56,57], as follows:

(10) $$D\left(i,j\right)=\{\begin{array}{cc} 0 & P\left(i,j\right)=C\left(i,j\right)\\ 1 & P\left(i,j\right)\neq C\left(i,j\right) \end{array},$$

here P(i,j) and C(i,j) are pixel values of the original and encrypted images, and L is the largest pixel value of both images. 

The values of both NPCR and UACI indicate the resistance of the encryption method against the differential attack.

#### 2.8.6. Information Entropy Analysis

Information entropy assesses uncertainty of a random variable, as follows [56]:

(11) $$E=\sum_{i=1}^{256}P\left(i\right)\log\left(\frac{1}{P\left(i\right)}\right),$$

here P(i) is the probability of the presence of pixel i. 

A larger entropy value indicates a greater level of security when applied to evaluate image encryption. Usually, an entropy value that is very close to a perfect value of 8 is considered to be safe from a brute force attack.

#### 2.8.7. PSNR Analysis

The Peak-Signal-to-Noise Ratio (PSNR) can be used to assess the quality of an image. A good image encryption method is expected to produce encrypted images with a low value of PSNR. Mathematically, PSNR is calculated by:

(12) $$PSNR=10\log_{10}\frac{255\cdot 255}{\sqrt{MSE}},$$

where P(i,j) is pixel value of the original image, and C(i,j) is pixel value of the encoded image, and Mean Square Error (MSE) is calculated as:

(13) $$MSE=\sum_{i=1}^{N}\sum_{j=1}^{M}\frac{{\left[P\left(i,j\right)-C\left(i,j\right)\right]}^{2}}{NM},$$

## 3. Results and Analysis

### 3.1. Settings

**Figure 4:**
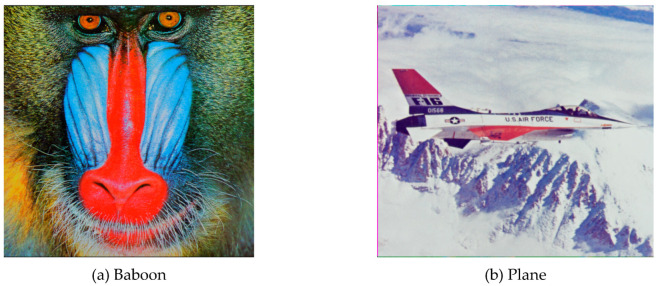

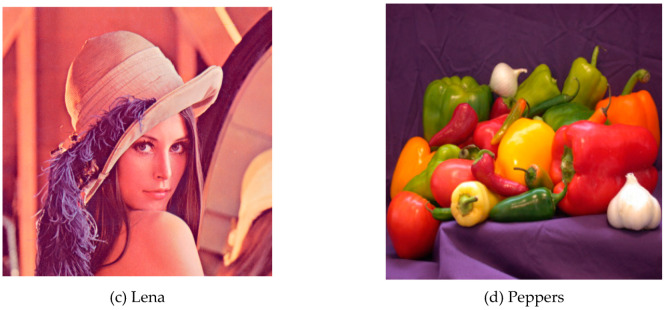
Original images: (**a**) Baboon, (**b**) Plane, (**c**) Lena, and (**d**) Peppers.

**Figure 5:**
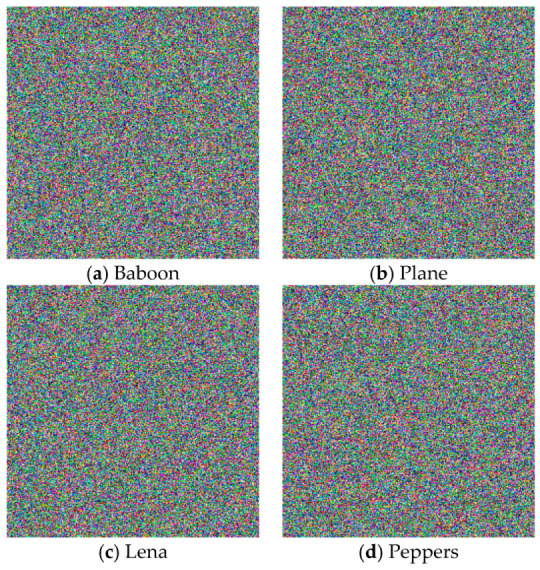
Encrypted images: (**a**) Baboon, (**b**) Plane, (**c**) Lena, and (**d**) Peppers.

All of the simulations were performed on a desktop computer with Intel ® Core™ i5-2430M CPU 2.4GHZ Processor, 4GB RAM, and Windows 8 Professional operating system. We used the freely available images from the USC-SIPI image dataset, such as Baboon, Plane, Lena, and Peppers, as the original protected images (see Figure 4). Figure 5 shows the encrypted images.

### 3.2. Results

**Figure 6:**
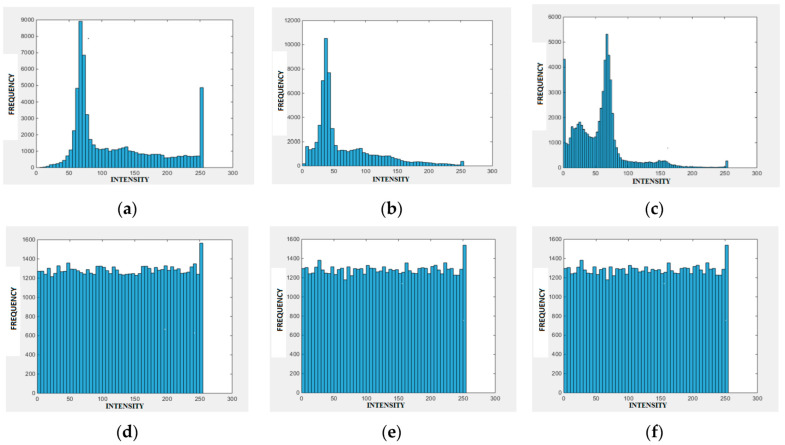
Histograms of Peppers image for red (**a**), green (**b**) and blue (**c**) channels, and histogram of an encrypted Peppers image for red (**d**), green (**e**), and blue (**f**) channels.

Standard techniques and tests are recommended for evaluating the results [51,52,53]. The quality of the proposed encoding method is indicated by the uniform distribution of pixel values in an encrypted image while using the histogram analysis method. As an example, see a histogram of the Peppers image for red (R), green (G), and blue (B) channels in Figure 6a–c. Figure 6d–f presents the RGB channels of the encrypted Peppers image. Here, the uniform distribution indicates that it would be difficult for the attacker to decipher the data.

**Figure 7:**
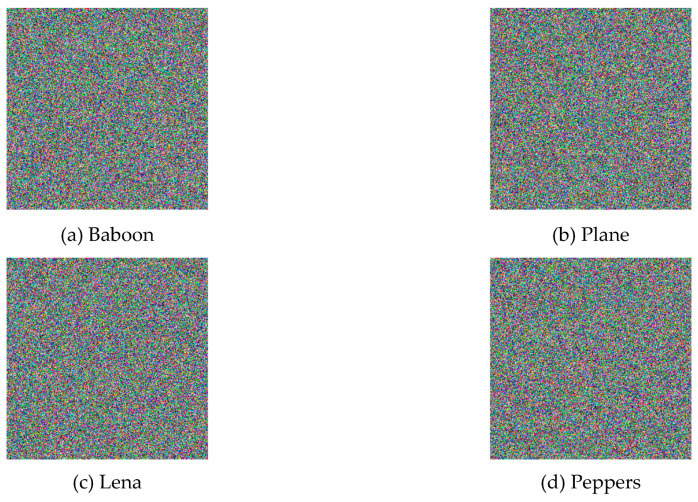
Decrypted images with correct secret key, but the initial value changed from $X_{0}=0.790$ to $X_{0}=0.791$: (**a**) Baboon, (**b**) Plane, (**c**) Lena and (**d**) Peppers.

It will not be possible to reconstruct the enciphered image if the keys differ by a small value [23]. As an example, Figure 7 shows the reconstructed images with a minor change of the secret key from $X_{0}=0.790$ to $X_{0}=0.791$, leading to totally different images. 

**Figure 8:**
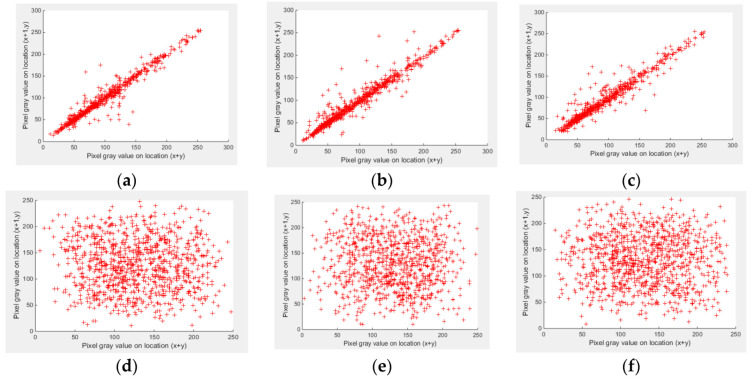
Relationship between neighboring pixels in horizontal (**a**), vertical (**b**), and diagonal (**c**) directions of plain Pepper image, and relationship between neighboring pixels in horizontal (**d**), vertical (**e**), and diagonal (**f**) directions of encrypted Pepper image.

The scattered graph can show the correlations between the neighboring image pixels. 1000 random neighboring pixels from an image are used to show the relationship. As an example, Figure 8a–c demonstrates a strong correlation between adjoining pixels, along horizontal, vertical, and diagonal neighboring pixels in the Peppers plain image. However, the correlation between the neighboring pixels is weak for an encrypted Pepper image (see Figure 8d–f). 

The correlation values for plain and encrypted images are given in Table 1 for the Lena, Peppers, Barbara, and Baboon images, along the horizontal, vertical and diagonal directions.

Table 2 presents the results of NPCR and UACI, PSNR, and Entropy. The values of NPCR and UACI demonstrate that the algorithm is very resistive against differential attack. The entropy is quite close to a perfect value of 8, and demonstrates that the proposed encryption method randomized the pixels in the encrypted image well. The obtained PSNR values are low, hence they also show that the proposed algorithm is good.

The proposed method is compared with other methods, while using entropy, NPCR, UACI, and correlation analysis for the Lena image of size 256 × 256 in Table 3. Here, we compare our method with other methods that were proposed by Li et al. [50] by Zhang et al. [27], Xu et al. [58], Wang et al. [59], Liu and Wang [60], Hussain et al. [61], Wang and Zhang [62], and Ahmad et al. [56]. 

In an occlusion attack, the enciphered image, which is transmitted over communication channels, could lose blocks of information. The robustness of the proposed algorithm against 12.5%, 25%, and 50% of occlusion in an encrypted image is evaluated while using MSE. The resulting MSE values are 1542.8362 for 12.5%, 3214.7971 for 25%, and 6927.9417 for 50% occlusion. We can claim that the proposed method can resist an occlusion attack, as the deciphered image still can be retrieved.

**Figure 9:**
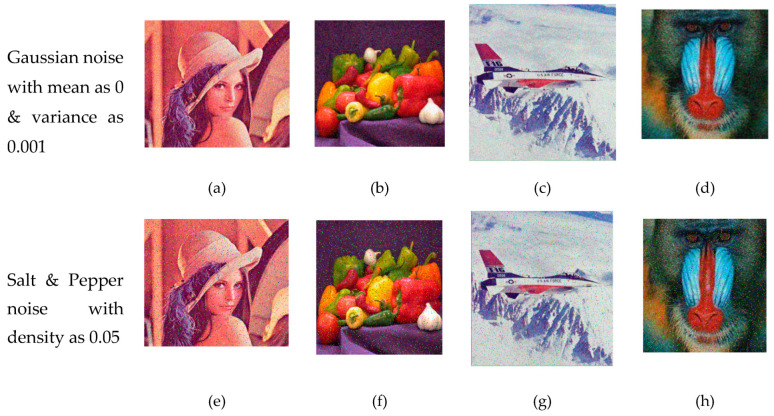
Analysis of noise attack: decrypted images after adding Gaussian noise, (**a**–**d**); decrypted images after adding salt and pepper noise (**e**–**h**).

Following the suggestion of Askar et al. [63], the original images are corrupted by adding Gaussian noise with the mean of 0 and variance of 0.001, as well as with salt and pepper noise with the density of 0.05. Figure 9 shows the obtained deciphered images. To compare, Table 4 shows the MSE and PSNR values. Based on Table 4, it can be concluded that the proposed method is resistant to salt and pepper noise, since the PSNR value exceeded 58 dB.

**Figure 10:**
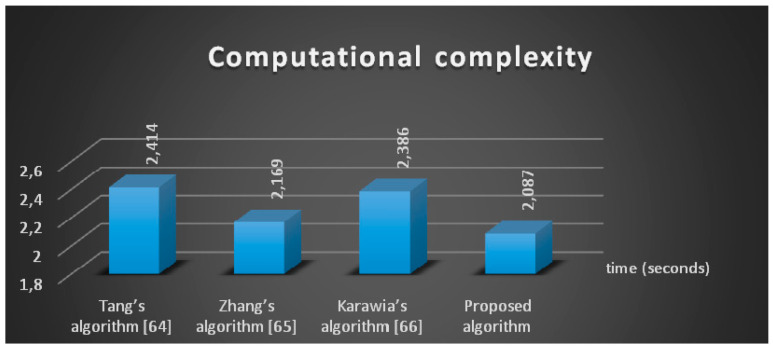
Comparison of computational time for the proposed algorithm.

The Tang’s algorithm [64], Zhang’s algorithm [65], and Karawia’s algorithm [66] were compared for the computational performance analysis. The results are shown in Figure 10 for the same set of four images, and they demonstrate that the proposed algorithm is computationally more efficient.

## 4. Statistical Test Analysis

The NIST statistical test suite (version 2.1.1, National Institute of Standards and Technology, Gaithersburg, MD, USA) [67] was used to evaluate the randomness of the bit sequence that was produced by the proposed system. The suite involves 15 tests, which assess the randomness that might occur in a series. Table 5 presents the results. Table 5 indicates that the NIST test is effectively performed: all p−values among 1000 sequences used for testing are evenly distributed in the 10 subintervals, while the pass rate is also acceptable. The average pass rate is 99.1% with a minimum pass rate of 98.4%.

## 5. Conclusions

We introduced an image encryption method that is based on a chaotic map with a new symmetric key generation system. The scheme uses Block Scrambling and Modified Zigzag Transformation, while key generation is performed using the enhanced logistic-tent map. Confusion and diffusion are achieved by pixel shuffling. It provides priority to resisting the brute-force attack to the suggested algorithm. The experimental results revealed that the suggested method has generated the encrypted images with uniform distribution in pixel histograms. Moreover, the suggested algorithm has shown that the encrypted pictures have information entropy of close to 8. It is able to robustly resist chosen/known plaintext attacks, is robust to salt and pepper noise, and it can withstand up to 50 percent occlusion attack. The comparison experiments were performed with other recent algorithms. The results of the statistical testing indicate that the new pseudo-random bit combiner can provide file encryption/decryption safety. We claim that the method is secure and computationally efficient based on the analysis of the proposed method. The proposed algorithm is simple, fast, and has strong practical application value.

## Figures and Tables

**Table 1:** Results of correlation analysis.

Images	Horizontal		Vertical		Diagonal	
	Plain	Cipher	Plain	Cipher	Plain	Cipher
Lena	0.9505	−0.0237	0.9745	−0.0237	0.9668	−0.0284
Peppers	0.9789	−0.0727	0.9750	−0.0225	0.9711	−0.0242
Barbara	0.9444	−0.0298	0.9555	−0.0611	0.9225	−0.0294
Baboon	0.9618	−0.0261	0.9686	−0.0572	0.9475	−0.0356

**Table 2:** Results of Number of Pixels Change Rate (NPCR), Entropy, Unified Average Change Intensity (UACI), and Peak Signal to Noise Ratio (PSNR).

Images	NPCR (%)	UACI (%)	PSNR	Entropy Plain Image Cipher Image	
Baboon	99.6017	33.2039	11.8337	7.2730	7.9993
Barbara	99.6073	33.5692	8.6936	7.6320	7.9990
Lena	99.6221	33.5887	6.7494	7.7329	7.9994
Peppers	99.5987	33.9060	9.8369	7.3785	7.9992

**Table 3:** Performance evaluation and comparison with other methods (best values are shown in bold).

Measure	[50]	[56]	[27]	[58]	[59]	[60]	[61]	[62]	Proposed
Horizontal correlation	0.0327	0.9407	**0.0018**	−0.0230	0.0020	0.0965	−0.0067	−0.0098	−0.0237
Vertical correlation	0.0219	−0.0273	0.0011	0.0019	−**0.0007**	−0.0318	−0.0137	−0.0050	−0.0178
Diagonal correlation	0.0180	−0.0140	**−0.0012**	−0.0034	−0.0014	0.0362	−0.0563	−0.0013	−0.0284
Entropy	7.9993	n/a	7.9994	7.9974	7.9970	n/a	n/a	7.9974	**7.9995**
UACI	n/a	15.38	33.4365	3.5100	27.97	n/a	33.4647	32.48	**33.5887**
NPCR	n/a	99.10	99.6166	99.6200	98.36	n/a	98.6810	93.21	**99.6221**

**Table 4:** Mean Square Error (MSE) and PSNR between input images and decrypted images distorted by adding noise.

Sample Images	Gaussian Mean = 0 & Variance = 0.001		Salt & Pepper Density = 0.05	
	MSE	PSNR	MSE	PSNR
Baboon	0.2698	53.8199	0.1711	58.7987
Plane	0.2711	53.7987	0.0793	59.1405
Lena	0.2013	54.1722	0.1022	58.0382
Peppers	0.2368	54.3872	0.0995	58.3375

**Table 5:** Results of NIST statistical test [67] for 1000 sequences, 1 million bits each, generated by the proposed scheme.

NIST Test	p-Value	Pass Rate
Frequency (monobit)	0.576884	995/1000
Block-frequency	0.783572	996/1000
Cumulative sums (Forward)	0.541882	996/1000
Cumulative sums (Reverse)	0.914691	993/1000
Runs	0.843905	984/1000
Longest run of Ones	0.062147	986/1000
Rank	0.400721	991/1000
FFT	0.186524	993/1000
Non-overlapping templates	0.497492	993/1000
Overlapping templates	0.230513	990/1000
Universal	0.087607	986/1000
Approximate entropy	0.198766	994/1000
Random-excursions	0.689012	615/621
Random-excursions Variant	0.397213	618/621
Serial 1	0.893692	992/1000
Serial 2	0.699795	993/1000
Linear complexity	0.344217	992/1000
